# Complex Network Study of Solar Magnetograms

**DOI:** 10.3390/e24060753

**Published:** 2022-05-26

**Authors:** Víctor Muñoz, Eduardo Flández

**Affiliations:** Departamento de Física, Facultad de Ciencias, Universidad de Chile, Casilla 653, Santiago 7800003, Chile; eduardo.flandez@ug.uchile.cl

**Keywords:** complex networks, sunspots, magnetograms, solar activity, complexity

## Abstract

In this paper, we study solar magnetic activity by means of a complex network approach. A complex network was built based on information on the space and time evolution of sunspots provided by image recognition algorithms on solar magnetograms taken during the complete 23rd solar cycle. Both directed and undirected networks were built, and various measures such as degree distributions, clustering coefficient, average shortest path, various centrality measures, and Gini coefficients calculated for all them. We find that certain measures are correlated with solar activity and others are anticorrelated, while several measures are essentially constant along the solar cycle. Thus, we show that complex network analysis can yield useful information on the evolution of solar activity and reveal universal features valid at any stage of the solar cycle; the implications of this research for the prediction of solar maxima are discussed as well.

## 1. Introduction

Studying solar dynamics is a subject of great interest, both for the observational, theoretical, and computational challenges involved and for the impact that solar magnetic activity has on our planet through the coupling of solar wind and the Earth’s magnetic fields, whereby periods of high solar activity may lead to intense geomagnetic storms which may affect human communications and spacecrafts [1]. Periods of low solar activity can be correlated with anomalously cold periods in the past [2], which is why many efforts have been devoted to the understanding or prediction of the solar cycle.

Several strategies have been developed to understand solar dynamics, including fluid and kinetic analytical models and computer simulations based on these models. In the last several decades, new strategies based on the study of complexity have been developed to study plasma systems. For instance, fractal and multifractal analyses have been carried out for turbulence in laboratory plasmas [3,4], geomagnetic activity [5,6,7,8,9,10], and solar wind dynamics [11,12,13]. From the perspective of self-organized criticality, sandpile models have been used to model the energy release from the Sun by means of solar flares [14] and geomagnetic activity [15].

Complex networks have proven to be a new and interesting approach to the study of plasma dynamics in general and of solar activity in particular. They have already been used in the context of various geophysical problems such as earthquakes, sea and atmospheric flows, and geomagnetic storms [16,17,18,19,20,21,22].

Regarding solar activity, complex networks have been successfully used to investigate time reversibility in turbulent states in solar wind simulations [23], to study probability of flares in solar active regions [24], to characterize the sunspots time series [25], and more recently to study solar flare statistics [26].

Most of these approaches consider complexity either in the spatial domain (e.g., fractal analysis of images) or in the time domain (e.g., fractality in time series and visibility graphs or nonlinear time series analysis in general). However, complex networks are able to follow the spatiotemporal evolution of a system as well, by mapping spatial patterns to nodes and time patterns to their connections. This strategy has been successfully applied to the study of seismic events, where it has revealed various universal features of seismicity regardless of the seismic zone [16,19,20] as well as changes in the network topology due to the existence of large events [27]. It has been further applied to the study of solar flares, where, combined with a visibility graph approach, it has shown scale-free and small-world features similar to those observed for networks based on seismic events.

In this paper, we take a similar strategy to studying solar activity by mapping the spatiotemporal evolution of sunspots on the solar surface to a complex network. As the sunspot number is regarded as a simple and effective way to describe the solar cycle, being directly related to the level of magnetic activity, changes in the number, distribution, and lifetime of sunspots may lead to changes in the topological properties of the resulting complex network, which can then be related back with the physical evolution of the solar magnetic field.

This paper is organized as follows. In Section 2, the dataset and analysis methods used to study them are described. First, in Section 2.1 the dataset used for this study is described. Then, the image recognition algorithms used to identify individual sunspots are described in Section 2.2. Next, Section 2.3 describes how a complex network was built from sunspot data, and the measures that were chosen to analyze it. Our results are provided in Section 3, and they are further summarized and discussed in Section 4.

## 2. Materials and Methods

### 2.1. Solar Magnetograms

Solar magnetograms are bidimensional representations of the magnetic field in the Sun’s photosphere. In this paper, we have taken data collected by the Michelson Doppler Imager (MDI) for the Daily Magnetic Field Synoptic Data (Solar Oscillations Investigation (SOI) project onboard the SOHO mission [28,29]). While each magnetogram is associated with one calendar day, they are actually averages over a 27-day solar rotation, thus representing the magnetic field magnitude on the solar disk. A typical magnetogram image can be found in Figure 13 of Ref. [5].

For this paper, we considered all magnetograms corresponding to the 23rd solar cycle, which started in July 1996 and ended in December 2008. The corresponding maximum of solar activity occurred in November 2001.

### 2.2. Image Processing Algorithms

Starting from the magnetogram images, we extracted information about sunspot location. First, we converted them into a black and white image, with white regions representing sunspots. Here, we follow the same procedure described in [5] involving conversion to gray scale. At this point, the image is represented by pixels which can have any value between 0 (black) and 255 (white). Then, a suitable threshold α is chosen such that all values above α are considered white and all values below α are considered black. In [5], the authors’ interest was to follow the evolution of the fractal dimension of the magnetograms, and the choice of α was therefore based on two criteria: that the resulting fractal dimension be able to actually discriminate stages along the solar cycle, and that the fractal dimension not be too sensitive to α (which is, after all, an arbitrary parameter). Based on that criteria, α=155 was chosen; we use the same value here for consistency. The results presented herein show that this method is able to discriminate between different stages of solar activity in complex network analysis.

Up to this point, the images are the same as those used for the fractal analysis in [5], with white dots indicating locations with higher magnetic field. However, in this work we intend to follow the spatiotemporal evolution of sunspots, and thus need to identify them, and their location, individually.

**Figure 1:**
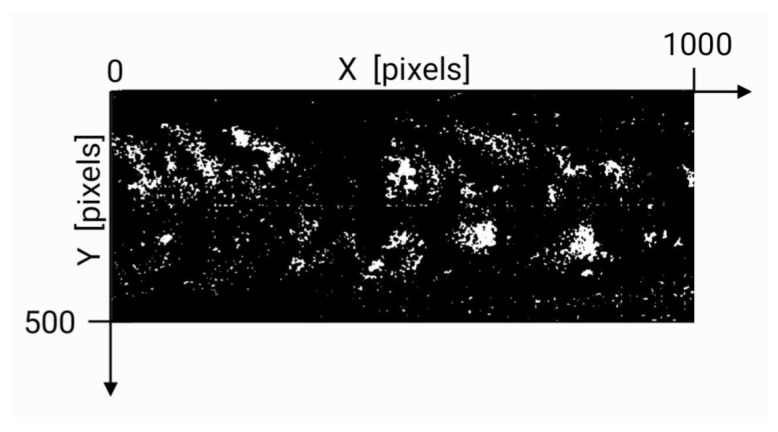
Filtered magnetogram for 1 January 2000; the axes show the coordinate system as used by the image processing algorithms described in the text.

Thus, we required additional image recognition and processing algorithms. First, we applied a noise reduction filter in order to eliminate isolated dots and focus only on major features which better represent actual sunspots. This was carried out by applying the MATLAB nlfilter function (https://la.mathworks.com/help/images/ref/nlfilter.html, accessed on 20 May 2022). By inspection, we determined that three passes of the filter were enough to eliminate noise and leave only relevant sunspots, yielding trends consistent with works reported elsewhere [30], as shown in Section 3. Figure 1 displays the resulting image after all the described steps, corresponding to the magnetogram for 1 January 2000. The original magnetogram and its conversion into a black and white pattern before noise filtering can be found in Figures 13 and 15 in Ref. [5].

Then, we used the MATLAB bwlabel function (https://la.mathworks.com/help/images/ref/bwlabel.html, accessed on 20 May 2022) to identify each sunspot as an independent object. Finally, the function regionprops (https://la.mathworks.com/help/images/ref/regionprops.html, accessed on 20 May 2022) was used to determine the respective centroids (in pixel coordinates).

As time evolves and as the Sun rotates, the centroids move, resulting in different coordinates for the centroids. In order to ensure that this phenomenon was not mistaken for two different events, we measured all centroid coordinates with respect to the origin of the Carrington coordinate system, which can be identified in the magnetograms and moves horizontally at a constant rate in consecutive magnetograms.

### 2.3. Complex Network

The set of centroids (pixels) for all magnetograms was used to build a complex network. Here, we follow previous work on seismic events [16,19,20,27,31], where seisms define the nodes and a node *A* is connected to a node *B* if the event associated with *B* is the one following the event associated with *A* in the seismic catalog. This approach has revealed interesting universal features for various seismic regions, although it is important to note that network edges do not imply causal relationships. For instance, it has allowed the universality of scale freedom and small-world features to be established in the resulting networks [27,31], as well as the universal behavior of the clustering coefficient regardless of the detailed features of the studied seismic region [19] and the change in network topology and critical indices before and after large seismic events [20]. These works suggest that complex networks built as described can be useful in studying physical systems by means of their spatiotemporal patterns. A similar idea, including a visibility graph approach, has been recently used to study solar flare statistics [26].

Here, then, we consider each centroid pixel as a node, and nodes are connected according to their time sequence. The main difference between our set of centroids and the seismic catalog is that at any given timestep there are several events (centroids) active, instead of only one. The simplest way to deal with this is to connect all nodes in the *n*-th magnetogram to all nodes in the n+1-th one.

Because sunspots may have lifetimes of several days, most of them appear in several consecutive magnetograms, which according to the previous rule should lead to several repeated connections between the same two nodes. As this is simply information about the simultaneous persistence of two nodes, we have ignored repeated connections between nodes; that is, two nodes can be connected, at most, by one edge.

Finally, as network construction requires a sequence of consecutive magnetograms, we must define time windows in order to use only those magnetograms which belong to it to build the complex network. We have found that non overlapping one-month windows are sufficient to reveal interesting features, as shown in Section 3.

In principle, as connections between nodes are forward in time, the resulting networks are directed. However, following [16,19,20,27,31], we can study the corresponding undirected networks by removing multiple connections between nodes and self-connections.

After the networks are built, various measures must be calculated in order to study their possible correlation with magnetic activity along the solar cycle. In particular, we considered node degree, clustering coefficient, mean path length between nodes, network density, and various centrality measures (betweenness, eigenvector, and closeness centrality). We calculated measures for each node and then averaged them in order to find the representative value for the whole network.

However, this average could be nonrepresentative if fluctuations are large; thus, a better description of the network should consider the distributions of these quantities. These distributions can provide interesting information about the underlying dynamics of the system. For instance, random networks yield exponential degree distributions, while a preferential-attachment growth model yields a power-law degree distribution [32]. In addition, decay exponents can change as the result of large events [20], and may correlate with the parameters of the network growth model [33].

Another way to analyze distributions is the Gini coefficient, which is typically used in economics as a measure of inequality in a society, providing an interesting abstraction of how a variable is distributed across individuals. This can be easily generalized to the distribution of a given measure across network nodes. Thus, based on the probability distribution function (PDF), we calculated the Gini coefficient in order to measure the “inequality” in the network representing the spatiotemporal pattern of sunspots.

## 3. Results

**Figure 2:**
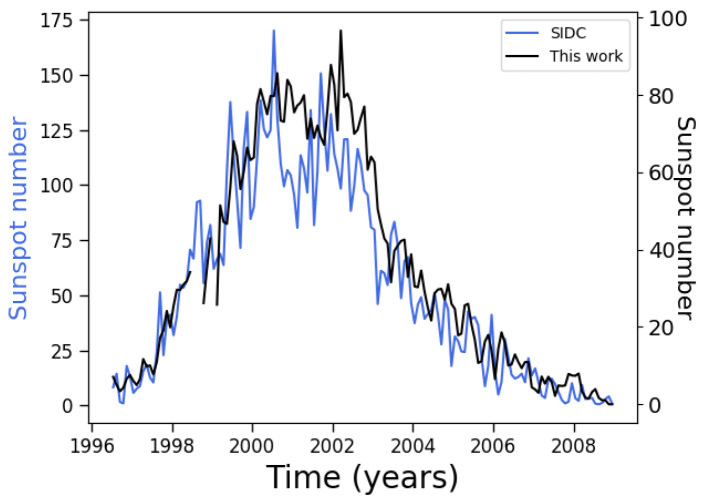
Sunspot number along the 23rd solar cycle as obtained by our algorithm. Black line: this work; Blue line: SIDC sunspot number.

As a first test of the quality of our procedure to isolate sunspots, we simply count the number of centroids in each magnetogram. Figure 2 shows the result obtained when three passes of the noise reduction filter are applied. The shape of the curve is consistent with previous works. For instance, Ref. [30] presents results obtained with the STARA code, which is based on image processing algorithms. Notice that they scale their results in order to compare with the international sunspot number reported by the Solar Influences Data Center (SIDC, [34]), as although absolute values are different, it is trends that are relevant. The same occurs in our case, where although our algorithms yield absolute values which are different from the SIDC number, the curves behave similarly. In effect, their Figure 1 shows that the trends are actually the same, as does our Figure 2. Notice that after 1 July 2015 a new data series is available for sunspot data at [34]. In order to compare with [30], the original series previous to 2015 has been used. Similar results are obtained with the revised dataset, except that a higher absolute number of sunspots is found in the latter. In particular, it is interesting that both the SIDC number and the STARA algorithm show a double-peaked maximum. However, the STARA algorithm shows a higher second peak. In that sense, our Figure 2, which is likewise based on image processing algorithms, is consistent with the result in [30] as well.

We note that around 1999 there is a lack of points in the curve. This is due to data collection problems in the spacecraft (loss of contact for several months following June 1998); thus, no magnetograms, or only low quality ones, exist for this period.

**Figure 3:**
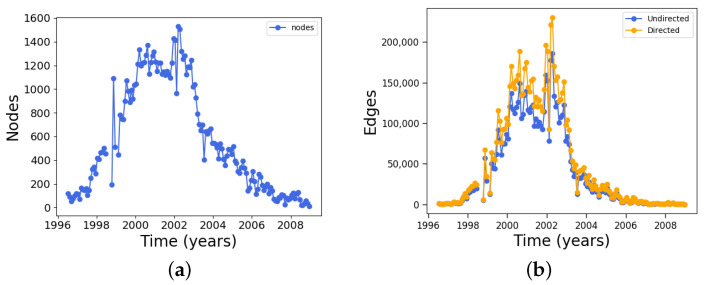
(**a**) Node number for one-month windows and (**b**) edge number for networks obtained for one-month time windows along the 23rd solar cycle.

We now use one-month windows to build the network, as described in Section 2, and calculate various measures, starting with the simplest ones. Figure 3a shows the number of nodes in one-month time windows, and Figure 3b shows the number of edges.

As expected, the curve for the number of nodes follows the trend of the sunspot number, because each node simply corresponds to a sunspot (except that the same sunspot may appear in several magnetograms, and thus the actual number of nodes is higher). A similar result is found for edges. As all nodes at time *t* are connected to all nodes at time t+1, it can be expected that when more nodes are present (near solar maximum), more edges will appear. While the number of edges is slightly decreased when the undirected network is considered because it involves removing edges from the directed network, the general trend is the same.

**Figure 4:**
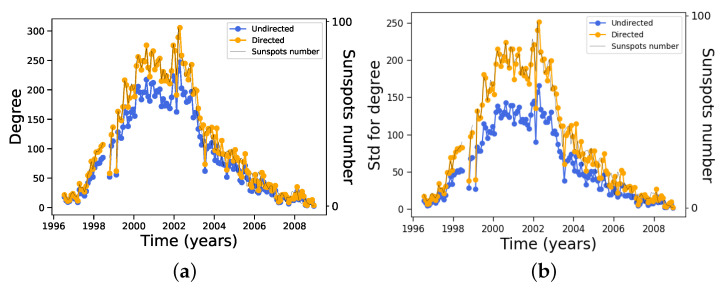
Measures for each monthly network along the 23rd solar cycle: (**a**) average degree and (**b**) standard deviation for degree.

Consistent with the results above, if both node and edge numbers increase during solar maximum we can expect the average degree of the network to increase as well. This is actually the case, as shown in Figure 4a. However, the increase in edge number as the solar maximum approaches induces a large increase in the fluctuations of the degree across the network. This can be noted in Figure 4b, where the standard deviation for the degree is plotted for both the directed and undirected networks. The same trend as all the other plots is observed. This suggests that although averages of a measure can show a good correlation with solar activity, deviations from the average may be very large, making the average itself less meaningful; thus, we must pay attention to the distribution of that quantity over the whole network as well.

In general, for the rest of the measures we have considered in this work their standard deviation does not have such extreme variations as for the degree distribution shown in Figure 4b, and they tend to be larger during solar minima instead.

**Figure 5:**
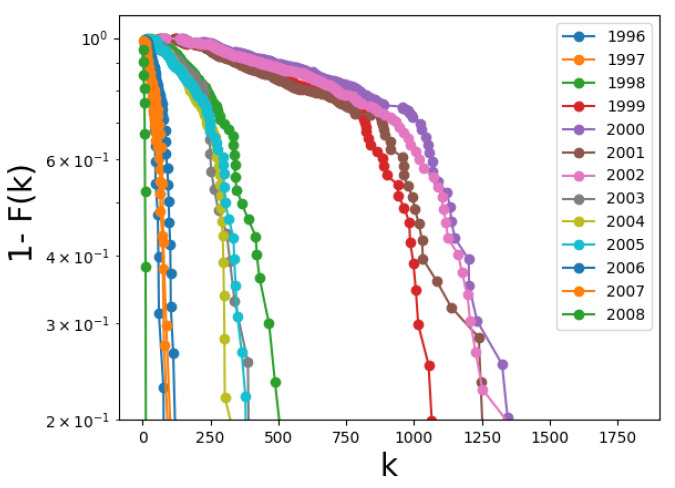
Complementary cumulative probability distribution function 1−F(k) for node degree, in semilogarithmic scale. All curves correspond to the month of July of the corresponding year.

Figure 5 shows the complementary cumulative probability distribution function (CCDF) for the degree for all years in the solar cycle. For the sake of comparison, we picked a particular network for each year, namely, the network for the month of July. We observe a clear difference between distributions corresponding to years near the solar minimum and solar maximum. Close to the solar maximum, larger degrees appear, causing both the average degree and its standard deviation to increase, as seen in Figure 4.

**Figure 6:**
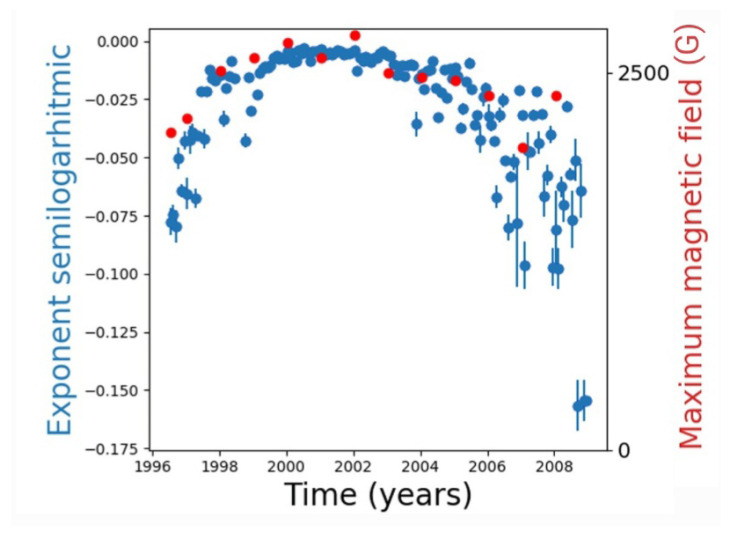
Decay exponent for the distributions in Figure 5 along the 23rd solar cycle; Blue dots: decay exponent for each monthly network; bars correspond to the error in the least squares fit; Red dots: Maximum magnetic field on the sunspot umbra, averaged per year [30].

Several previous works have shown that the decay features of the distribution function may yield information on the underlying physics leading to network formation [32,35], such as optimization mechanisms for the growth of spatial networks [33,36] and revealing the existence of large earthquakes in networks of seismic data [20]. Based on this, we turn our attention to the tail of the distributions. In Figure 5, there seems to be an exponential decay with a varying decay exponent as the solar cycle progresses. This is explicit in Figure 6, where the time evolution of the exponent is shown (blue dots). It is interesting to compare these results, which are derived from an essentially structural analysis (complex network measure), with the physical parameters representing solar activity. This is shown in Figure 6 in the red dots, which correspond to the maximum magnetic field on the sunspots’ umbra, averaged per year, as reported in [30]. We think that the good correlation between the degree exponent and the sunspots’ magnetic field is nontrivial, and suggests that this network analysis can provide interesting information on solar activity.

**Figure 7:**
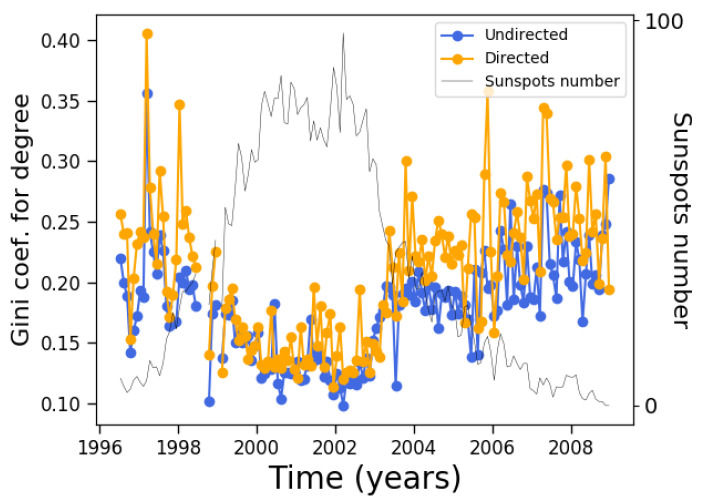
Gini coefficient for each monthly network along the 23rd solar cycle. For reference, the sunspot number as shown by the black curve of Figure 2 is plotted as well.

As mentioned above, the Gini coefficient is another measure that can be used to visualize how a given quantity is distributed across nodes, in addition the much simpler information provided by the average and standard deviation and without the need to deal with the distribution as a whole. In this case, we calculate the Gini coefficient for the degree distribution in order to obtain an estimation of how “unequally” the degree is distributed across nodes. The results for both the directed and undirected networks are shown in Figure 7.

It is clear from Figure 7 that the Gini coefficient decreases during solar maxima. In effect, during solar minima there are few sunspots, which leads to a low number of both nodes and connections. Not all nodes have the same degree, and thus there is a certain inequality in its distribution at the start of the cycle. However, as the solar cycle advances, the sunspot number increases, and the number of nodes and connections increases. Given the growth rules, connections are created between all nodes in consecutive magnetograms, leading to the possibility of decreasing inequality.

Notice that this trend is opposite to that of the average degree and standard deviation shown in Figure 4. It is worth pointing out that the difference between directed and undirected networks is much less for the Gini coefficient than it is for the degree and standard deviation.

To this point, we have found that certain measures correlate with solar activity (degree and standard deviation of the degree), while others anticorrelate (Gini coefficient). This is a consequence of the sunspot number evolution in time and space and the way the network is built, which yields new connections which are distributed in such a way that degree is more “equally” distributed across nodes when sunspot number increases. Thus, it is interesting to find that this interplay is able to yield measures which are essentially constant along the solar cycle.

**Figure 8:**
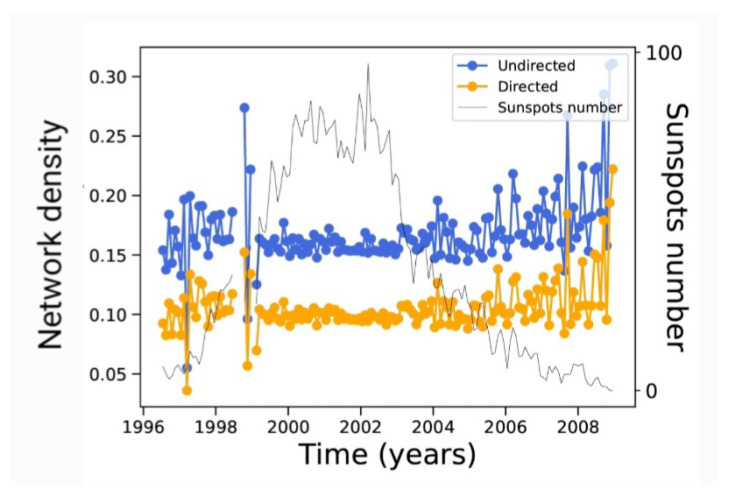
Network density for each monthly network along the 23rd solar cycle. For reference, the sunspot number as shown by the black curve of Figure 2 is plotted as well.

This is the case for the network density (the ratio of the edge number to the maximum possible number if every node were connected to all other nodes); network density is shown in Figure 8.

As seen in this plot, the undirected network is slightly denser that the directed one, which is consistent with it having more connections for the same number of nodes. However, the dynamics of new connections between nodes is such that density is essentially constant along the solar cycle. Fluctuations, meanwhile, are larger at solar minima, possibly due to the smaller number of connections and the resulting poorer statistics.

Thus far, we have only discussed results directly based on the degree of the nodes, which is certainly the simplest of the possible measures. We now discuss other more complex measures that allow to describe the network topology in complementary ways.

**Figure 9:**
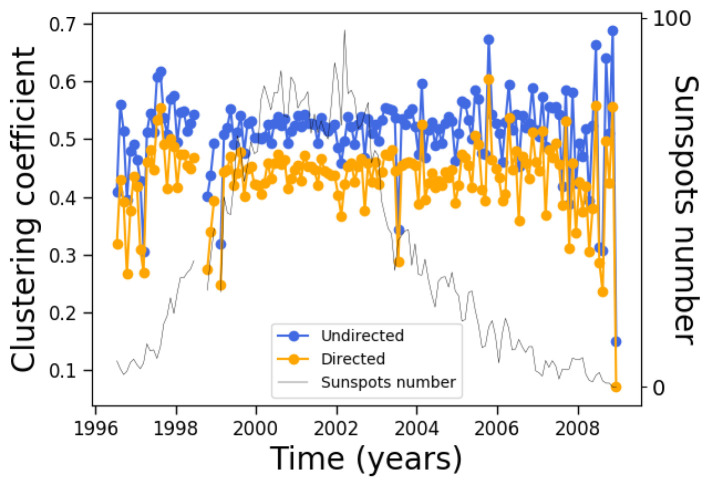
Same as Figure 8, except for the clustering coefficient.

That is the case for the clustering coefficient, which quantifies the number of neighbors of a given node which are in turn connected to each other. The result is shown in Figure 9.

A very interesting picture now arises. In principle, it would be reasonable to expect that the quantities measured on the complex networks would follow the solar cycle. As mentioned above, this means that Figure 3 and Figure 4 can be easily understood. However, not all measures depend in a trivial way on the number of nodes and edges; this is related to the topology of the network, as provided by both the number, location, and persistence of the sunspots (which provides the physics behind the process), and to the algorithm used to build the network. Figure 9 shows precisely this, as the clustering coefficient is a measure that depends on how neighbors are connected, which is not automatically related to their number. In fact, the clustering coefficient is essentially constant along the solar cycle. As this measure is provided by the number of closed triangles in the network, it is related to the number of possible connections between a given subset of nodes. Therefore, one can argue that Figure 9 is consistent with Figure 8. Notice that fluctuations of the clustering coefficient are larger at solar minima, which is possibly related to the poorer statistics available with the smaller number of nodes. The clustering coefficient is slightly larger for the undirected network, which is consistent with its larger density.

**Figure 10:**
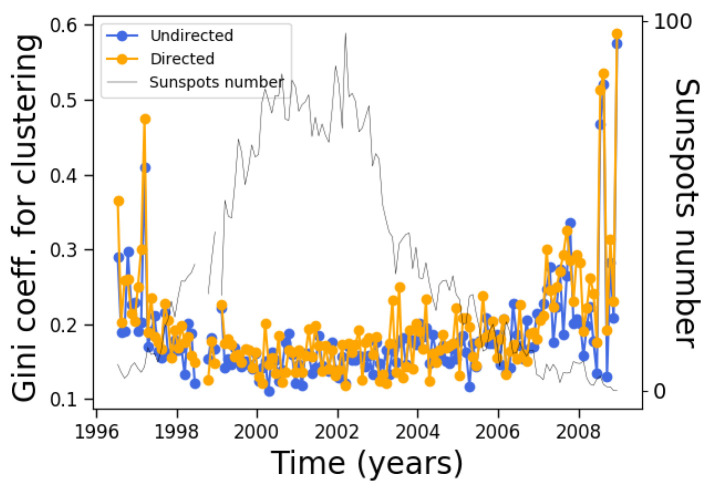
Same as Figure 8, except for the Gini coefficient of the clustering coefficient distribution.

On the other hand, although the average clustering coefficient does not change very much along the solar cycle, its distribution clearly changes, as manifested by its Gini coefficient (Figure 10). During solar maxima, the clustering coefficient is distributed more equally across nodes, similar to the degree (Figure 7).

We can now observe that these two quantities tell different stories along the solar cycle. The degree correlates with the solar cycle, while its inequality anticorrelates; on the other hand, the clustering coefficient does not correlate with the solar cycle, while its inequality anticorrelates.

**Figure 11:**
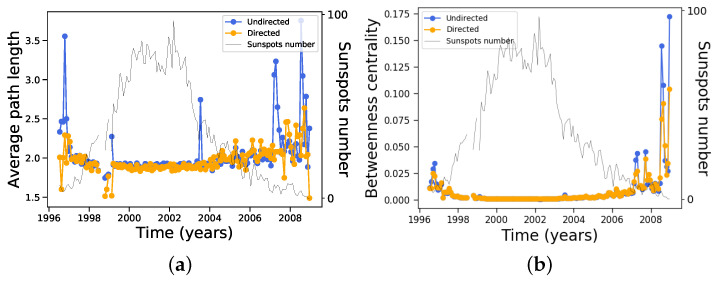
Same as Figure 8, except for (**a**) distance and (**b**) betweenness centrality.

Mean distance (length of shortest path) between nodes and betweenness centrality does not show any particularly interesting features, remaining essentially constant along most of the solar cycle except very close to solar minima, as shown in Figure 11.

**Figure 12:**
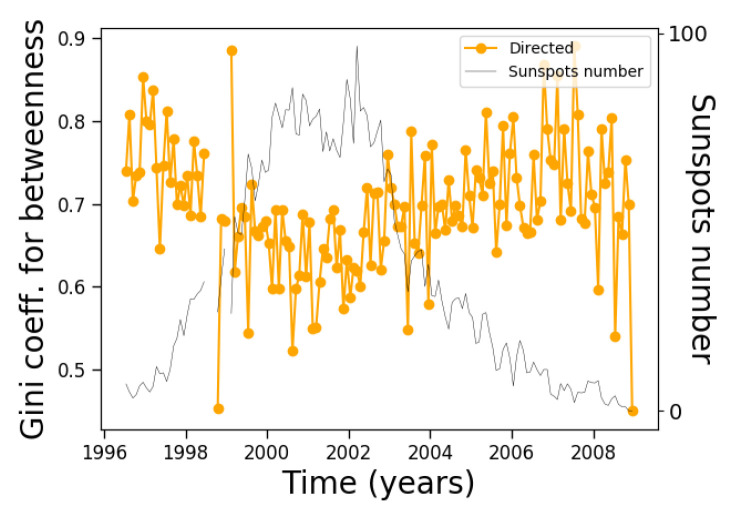
Same as Figure 8, except for the Gini coefficient of the betweenness centrality distribution.

However, as a further example of the nontriviality of the results and of the relationship between different measures, the Gini coefficient of the betweenness centrality (Figure 12) shows an interesting evolution along the solar cycle, oscillating between 0.5 and 0.9, approximately, with one minimum near the solar cycle maximum and a maximum before the final solar cycle minimum.

**Figure 13:**
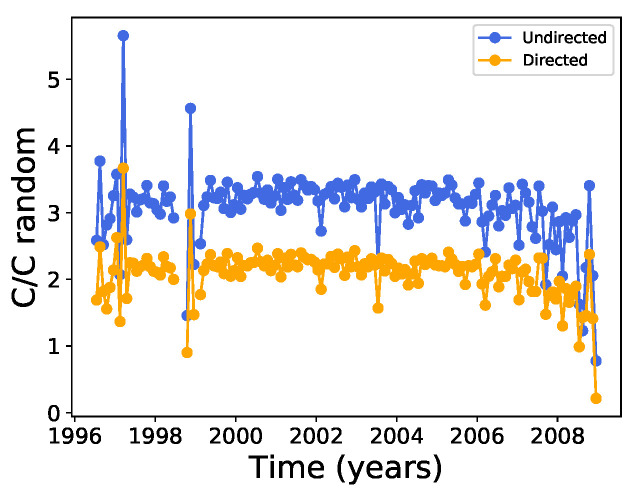
Same as Figure 8, except for the ratio between the clustering coefficient and the clustering coefficient of a random network with the same number of nodes and edges averaged over ten random instances.

The mean distance between nodes is of the order of 2 along the solar cycle, which is very small compared with the number of nodes (Figure 3), suggesting that the network may be of the small-world type [37]. In order to test this, we can calculate the ratio between the clustering coefficient of the network (Figure 9) and the clustering coefficient $C_{\mathrm{random}}$ of random networks with the same number of nodes and edges for each month along the solar cycle; thus, we improved these statistics by building ten different random networks to obtain an average value for $C_{\mathrm{random}}$, with the results shown in Figure 13. It can be seen that although the clustering coefficients are larger than those for the corresponding random network, the condition $C\gg C_{\mathrm{random}}$ is not satisfied, and small world features could therefore not be identified.

**Figure 14:**
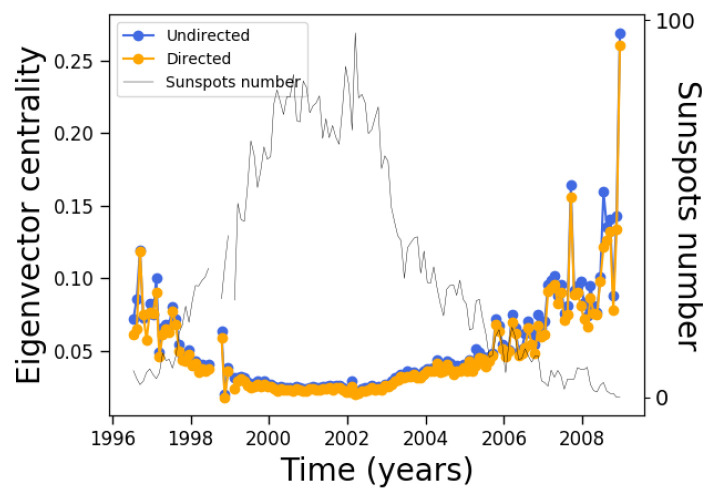
Same as Figure 8, except for the eigenvector centrality.

We made similar analyses for two additional measures of centrality, namely, closeness and eigenvector centrality. The closeness centrality shows similar behavior as the distance centrality and betweenness centrality (Figure 11), being approximately constant during most of the cycle. However, unlike Figure 11, the closeness centrality diminishes near solar minima, which makes sense when considering that this measure is related to the inverse of the distance between nodes. As for the eigenvector centrality, its evolution is similar to Figure 11, although with a more gradual increase near solar minima which makes the anticorrelation with solar activity much more clear (Figure 14).

**Figure 15:**
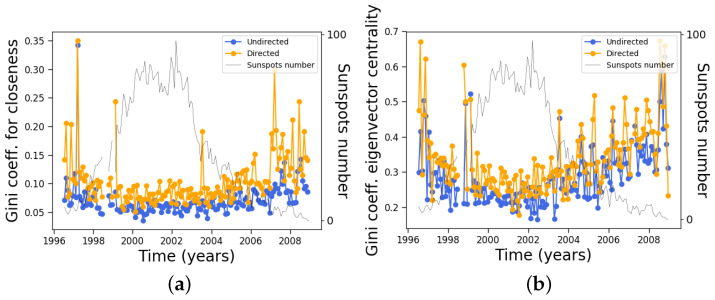
Same as Figure 8, except for the Gini coefficients of (**a**) closeness and (**b**) eigenvector centrality.

Regarding their respective Gini coefficients, the behavior is similar to the Gini coefficient for the clustering coefficient, anticorrelating with solar activity, although its variation along the solar cycle is much more evident for the eigenvector centrality. This is shown in Figure 15.

Several of the correlations between these measures and solar activity (as represented by the number of sunspots) can be easily explained, e.g., the degree, while others are less evident, such as the Gini coefficients and the decay exponents of the degree distribution. There are relationships between certain measures which can be expected; we have already mentioned that the density and the clustering coefficient (Figure 8 and Figure 9) are constant along the solar cycle, as they both compare the number of edges with the number of possible connections between a certain subset of nodes. We have pointed out that the inverse relation between the closeness centrality and the distance between nodes may explain the inverse convexity in their respective plots.

In order to reveal further and less obvious relationships between measures, we provide various plots where the abscissa correspond to values of one measure for a given network and the ordinate corresponds to the value of another measure for the same network. If the two measures selected are completely uncorrelated, the points should cover the plane randomly. If there is a strong linear correlation between the measures (revealing a possibly trivial dependence between their values), the points would lie on a single line. Notice that we consider measures which can be calculated for each single node (degree, clustering coefficient, centrality measures); thus, the plotted values are actually averages over all nodes for a certain monthly network, consistent with Figure 4, Figure 9, Figure 11b and Figure 14.

**Figure 16:**
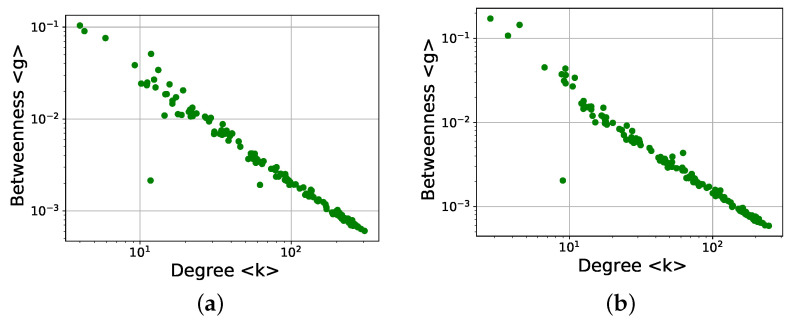
Average betweenness centrality versus average degree for monthly networks along the 23rd solar cycle: (**a**) directed networks and (**b**) undirected networks.

Figure 16 shows the betweenness centrality versus the degree. A clear power-law behavior is observed for both the directed and undirected networks:

(1) $$\langle g\rangle \left(\langle k\rangle \right)≃{\langle k\rangle }^{-\gamma }\;$$

with slightly different decay exponents (γ∼1.14 for directed networks and γ∼1.18 for undirected networks).

This is an interesting result. First, the plots for degree (Figure 4) and betweenness centrality (Figure 11b) do not suggest an evident relationship between both measures, which is clear in Figure 16. Second, various works have discussed the power-law relationship between betweenness centrality and degree for scale-free networks, including the possible universality of the decay exponent [27,38,39], and thus Figure 16 could be showing the scale-free nature of the networks, which is not otherwise evident (in Figure 5, for instance, we were able to fit the distributions with an exponential function). However, these works deal with a single network, and the values of betweenness centrality and degree for individual nodes are compared. Here, each point is provided by values representative of a complete network, and further work is therefore required in order to understand the implications of this result.

Notice that because Figure 4 shows that lower degrees occur close to solar minima and higher degrees close to solar maxima, in Figure 16 the points on the left side of the plot correspond to the beginning and end of the cycle, while the points on the right side correspond to the networks near the solar maximum.

In Figure 16, certain points deviate from the power-law relationship. For the directed networks, there are two outliers below the main group. These points correspond to March 1997 ($\langle k\rangle ≃10^{1}$) and February 1999 ($\langle k\rangle ≲10^{2}$). For the second case, February 1999, the problem is the quality of the magnetograms themselves, as mentioned above when discussing Figure 2, which affects the topology of the resulting network, deviating it from the general trend. For March 1997 the problem is less evident, because the quality of the magnetograms is better, except for the existence of a band without data in the upper part of the magnetogram. Although similar features can be observed in other parts of the solar cycle, March 1997 is in the minimum of the cycle; fewer sunspots are visible, and a moderate perturbation in the data can therefore yield strong effects in the network as a whole.

For the undirected networks, the major outlier for March 1997 remains, and the same explanation applies.

It is interesting to note that although degree and betweenness centrality correlate in different ways with solar activity, their respective Gini coefficients are not very different during most of the solar cycle, with slightly different trends at the end of the solar cycle (Figure 7 and Figure 12). Further analyses are needed to understand whether this fact and the correlation observed in Figure 16 is accidental or could be explained by more general arguments.

**Figure 17:**
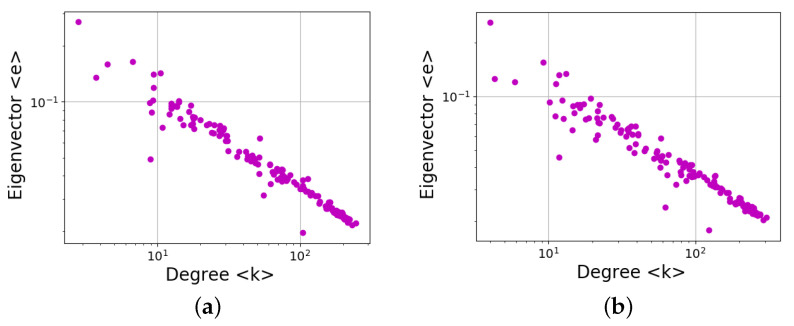
Same as Figure 16, except for the eigenvector centrality: (**a**) directed networks, (**b**) undirected networks.

Equivalent results are found for other centrality measure, namely eigenvector centrality. The results are shown in Figure 17, where a power-law dependence is again evident:

(2) $$\langle e\rangle \left(\langle k\rangle \right)≃{\langle k\rangle }^{-\delta }\;.$$

Similar to the betweenness centrality, the decay exponent in this case is only slightly different for directed and undirected networks (δ∼0.48 and δ∼0.5, respectively). It could be argued that this is to be expected, as the betweenness and eigenvector centrality show similar trends along the solar cycle (Figure 11b and Figure 14), although with a more clear dependence on the solar cycle for the latter.

However, unlike the betweenness centrality, the similarities between the Gini coefficients of the degree (Figure 7) and eigenvector centrality (Figure 15) are much closer than with the betweenness centrality (Figure 12), confirming that the power-law relationship between measures does not imply correlation between the distribution inequalities.

In Figure 17, as in Figure 16, there are clear outliers, which in this case correspond to March 1997 ($\langle k\rangle ≃10^{1}$), February 1999 ($\langle k\rangle ≲10^{2}$), and November 1998 ($\langle k\rangle ≳10^{2}$), the explanations being the same as above. The new outlier, November 1998, has problems in the data similar to February 1999, which are revealed by the choice of a different measure.

**Figure 18:**
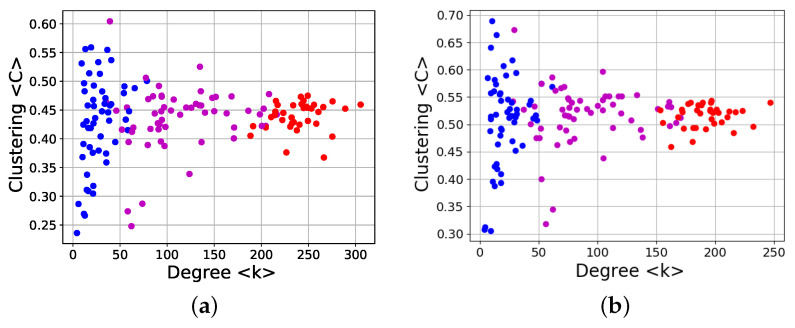
Same as Figure 16, except for the clustering coefficient. Colors identify stages in the solar cycle. Blue dots: years near solar minimum (1996–1997, 2006–2008); purple dots: years before or after solar maximum (1998–1999, 2003–2005); red dots: years near solar maximum (2000–2002). (**a**) Directed networks, (**b**) undirected networks.

Finally, we show results for the clustering coefficient versus degree, in Figure 18.

The plots are clearly different from Figure 16 and Figure 17, as no simple power-law dependence is observed; however, the distribution is not fully random either. This is especially evident when we add the information about the location of the corresponding time window in the solar cycle, represented by the different colored dots in Figure 18. It can be observed that the clustering coefficient is not fully independent of the degree, and the details change along the solar cycle. Near solar maxima the clustering coefficient is less correlated with the degree, as shown by the larger dispersion of blue points. At this stage, the small number of sunspots leads to fewer nodes and connections (Figure 4), meaning that these dots are located at the lowest values of the degree. The opposite occurs during solar maxima. The clustering coefficient exhibits the lowest dispersion, and as the sunspot number is larger at this time, the degree is the highest as well. Then, at intermediate stages of the solar cycle (ascending and descending phases) far from both the maximum and the minimum of solar cycle, both the dispersion of the clustering coefficient and the average degree have intermediate values. These results are independent of the network being directed or undirected. It is worth noting that it is the dispersion of the clustering coefficient which has an interesting evolution along the solar cycle, whereas its average value is essentially constant, as can be seen in Figure 18 and is explicit in Figure 9. This is consistent with the fact that the Gini coefficient of the clustering coefficient, Figure 10 is better correlated with solar activity than with the clustering coefficient itself.

## 4. Discussion

In this work, we have carried out an analysis of magnetic solar activity following a complex network approach. The complex network was built by associating nodes to sunspots and linking sunspots in consecutive days with edges. The study is based on magnetic field information contained in daily magnetograms representing the magnetic field intensity on the solar surface along the full 23rd solar cycle. The magnetograms were converted to black and white images, the noise was reduced, and image recognition algorithms were used to identify the centroids of individual sunspots, which in turn define the network nodes.

As the solar activity varies, the number and lifetime of sunspots change, and this should have an effect on the topology of the resulting network. This was tested by evaluating several measures for networks built with data corresponding to moving one-month windows along the solar cycle. Changes in the measures can then be expected to reveal associated changes in the topology of the network as a result of variations in solar magnetic activity.

Several measures were calculated, namely, degree, clustering coefficient, density, distance between nodes, betweenness centrality, and eigenvector centrality. Their average values were studied along with their full distributions and the associated Gini coefficients. We observe that certain quantities correlate and others anticorrelate with solar activity, as measured by sunspot number, whereas others are essentially constant along the solar cycle. This is already an interesting result, as certain measures (such as the degree) should obviously correlate with the solar number because they depend in a straightforward manner on the number of nodes and edges. However, the fact that other measures (e.g., clustering coefficient) do not change along the solar cycle is less trivial to explain.

The above is discussed in terms of the average values of the measures over the network nodes. As fluctuations around average values can be very large (for instance, at solar maximum both the average degree and its fluctuations increase; see Figure 4), we consider the full distribution of the measures. In the case of the degree, this yields an interesting result, as distributions are clearly different at solar maxima and minima. In fact, an exponential fit can be applied to the degree distribution; its decay exponent closely follows the curve of maximum values of the magnetic field on the solar surface (Figure 6).

This suggests that the topology of the complex network actually carries nontrivial information about the physical state of the system.

Further analysis shows that the Gini coefficient is a useful way to describe the distribution, beyond the simple information given by averages, as it provides information about the “inequality” of the distribution of a certain variable across nodes. In general, we find that all of the Gini coefficients that we calculated tend to decrease during solar maxima, regardless of the behavior of the averaged variable itself. This can be observed in Figure 7, Figure 10, Figure 12 and Figure 15. Thus, while average variables may correlate, anticorrelate, or be approximately constant along the solar cycle, the inequality in their distribution always tends to decrease during solar maxima. That is, the increase in nodes and edges has the effect of homogenizing the distributions rather than further concentrating them in a few already-prominent nodes. Although this result may be intuitive for quantities directly related to the number of nodes and edges, such as the degree, it is much less obvious for more elaborate measures such as the clustering coefficient and centrality measures. Nevertheless, these results seems to be universal for all measures studied.

These findings certainly point out the need to explore various measures in order to properly study the topology of a given network, as they all carry different complementary information. In our case, we have followed the evolution of measures along the solar cycle, as we expect that the changes in the number and lifetime of nodes lead to changes in the topology of the resulting networks. Thus, it is interesting (a) to find that this expectation is actually met with respect to several measures, (b) to notice that certain measures exhibit anticorrelation rather than correlation, and (c) to realize that although the average of a measure may not correlate, the full distribution does have useful information on solar activity, as shown by the Gini coefficients. It is interesting to note that it is possible to relate the decay exponent of the distribution, which is a quantity related to the network abstraction of the system, with a physical quantity such as the magnetic field in sunspots (Figure 6), further pointing out about the possible usefulness of the complex network approach for describing the magnetic evolution of the Sun.

As we have mentioned, the behavior of certain quantities is easier to understand, while in other cases the explanation is less trivial; although each measure provides a particular information of the system and they are independent of each other, there may be correlations between them, especially because we made decisions as to how the network is built which constrain the phase space for the measures, and particular correlations may therefore appear.

This is, for instance, the case for the density and the clustering coefficient, which are both related to the number of actual edges compared to the total number of possible edges between sets of nodes and which present similar behavior, essentially constant along the solar cycle (Figure 8 and Figure 9). The same occurs for the average distance between nodes and the closeness centrality, which are related to the inverse of the distance between nodes.

Less evident correlations were found by plotting one measure as a function of another measure. In effect, both betweenness and eigenvector centralities exhibit a power-law dependence with respect to the degree. This resembles previous findings for scale-free networks [27,38,39], although here each point represents a whole monthly network whereas the cited works involve measures for individual nodes. We believe that further work is needed in order to understand the consequences and meaning of these results, as it was not clear when examining the degree distribution that the networks are actually scale-free (Figure 5).

The nontriviality of the result above is highlighted by the fact that it is not universal, as shown for the clustering coefficient (Figure 18). However, the distribution of points is not random either; it is clear that the network clustering coefficient has the largest dispersion during solar minima and the smallest at solar maxima. We note that in all cases the average clustering coefficient is approximately constant, which is consistent with the Gini coefficient (Figure 10) being anticorrelated with magnetic solar activity while the clustering coefficient is not (Figure 9).

In this work, we have found that the complex network approach can be a useful approach to studying solar magnetic activity, as certain measures show correlations or anticorrelations with the sunspot number, which is a common way of following the solar cycle. In addition, it is possible to establish a connection between complex network, topological measures, and physical quantities such as the solar magnetic field along the solar cycle.

This opens the possibility of using this tool to make predictions of the solar cycle. In effect, various proposals are available to model various features of the solar cycle, such as the magnetic field evolution [40,41] or the number of sunspots [42,43], in order to predict parameters such as the time of occurrence and amplitude of the next solar maximum. Considering our results, could a complex network approach contribute to this question by providing a novel way to analyze the sunspot time series? We plan to address this question in future work.

We have further shown that not all measures are equally useful for this purpose, as not all are sensitive to the level of solar activity provided by the spatiotemporal pattern of sunspots. Therefore, this suggests useful candidate measures for either the description or the prediction of the solar activity. On the other hand, it is interesting that certain measures do not seem to vary along the solar cycle; further work should be done in order to understand to what extent the method used to build the complex network is responsible for this, as well as to understand what it can tell us about sunspot evolution and associated network growth.

We have shown that the differences between directed and undirected networks are marginal. All our conclusions hold for both types of networks, the only differences being in the actual numerical values of the measures.

It would be interesting to carry out similar studies for other solar cycles, in order to establish possible universal behaviors as well as particularities of each solar cycle. This would allow for testing of the eventual usefulness of this approach in making predictions of the following solar maximum. This is specially relevant because of the various anomalies present in solar cycle 23 with respect to other recent cycles [44,45], violating the empirical even–odd rule [46]; it was unusually long (12.4 years), and during the following minimum solar indices were exceptionally low [47]. However, it should be noted that the even–odd rule may have been violated in the past, and thus cycle 23 may not be anomalous on larger timescales [44]. Furthermore, these anomalies suggest a possible transition of the Sun to a new regime, forcing strategies previously used to describe the solar cycles to be revised [47]. In this sense, new tools, such as the one proposed here based on a complex network approach, may enrich the discussion.

Other algorithms for building such networks and for using magnetic field data instead of already-processed images are planned for future works; however, we believe this work may contribute, as other recent works have [24,25,26], to establishing complex networks as a useful approach to studying the evolution of solar magnetic activity.

## Data Availability

Publicly available datasets were analyzed in this study. Solar magnetograms, on which the analysis was performed, can be found at the webpage of the Solar Oscillations Investigation project at the following URL: http://soi.stanford.edu/magnetic/index6.html, accessed on 20 May 2022.
